# AST-to-Platelet Ratio Index as Potential Early-Warning Biomarker for Sepsis-Associated Liver Injury in Children: A Database Study

**DOI:** 10.3389/fped.2019.00331

**Published:** 2019-08-21

**Authors:** Jiaying Dou, Yiping Zhou, Yun Cui, Min Chen, Chunxia Wang, Yucai Zhang

**Affiliations:** ^1^Department of Critical Care Medicine, Shanghai Children's Hospital, Shanghai Jiao Tong University, Shanghai, China; ^2^Department of Information Technology, Shanghai Children's Hospital, Shanghai Jiao Tong University, Shanghai, China; ^3^Institute of Pediatric Critical Care, Shanghai Jiao Tong University, Shanghai, China

**Keywords:** APRI, liver injury, sepsis, early-warning, child

## Abstract

**Background:** Sepsis-associated liver injury (SALI) is a risk factor of poor outcome in patients with sepsis. The early warning biomarkers for identifying SALI remain poorly defined.

**Aims:** To identify the potential predictors of occurrence of SALI in pediatric patients with sepsis.

**Methods:** We retrospectively analyzed the sepsis database based on the medical records of patients admitted to the pediatric intensive care unit (PICU) in Shanghai Children's Hospital from July 2014 to June 2018. Patients' demographics, co-morbidities and laboratory variables were collected. Univariate and multivariate logistic analysis were used to explore risk factors of SALI, and receiver operating characteristic (ROC) curve analysis was used to evaluate their predictive significances for SALI occurrence.

**Results:** Of 1,645 eligible patients, 1,147 patients were included, and 105 cases had SALI. The indexes including AST-to-platelet ratio index (APRI), γ-GT and lactate dehydrogenase (LDH) were independent risk factors for SALI. Moreover, APRI was powerful to predict SALI in children (AUC: 0.889, 95% *CI*: 0.851–0.927) with a sensitivity of 84.6 % and a specificity of 84.3 % at the cutoff point of 0.340. APRI was superior to LDH and not inferior to γ-GT for predicting SALI.

**Conclusion:** APRI is an independent risk factor of SALI occurrence, and elevated APRI within 24 h after PICU admission (>0.340) is a potential predictor for SALI in children.

## Introduction

Sepsis is a leading cause for admission to pediatric intensive care unit (PICU), and it is life-threatening organ dysfunction caused by dysregulated host response to infection (1). Sepsis-associated liver injury (SALI) is one of the main clinical performances, which is an independent risk factor for multiple organ dysfunction and high mortality rate in pediatric patients with sepsis (2, 3). Preventable mortality due to SALI has been attributed to failures of early or accurate recognition, especially in children. Early warning and appropriate treatment of liver injury is critical for recovery in patients with sepsis.

The pathophysiology of liver injury is complex and not yet well-understood. Infection or shock, systemic inflammatory response, persistent microcirculatory failure, or even undesirable side effects of the treatment provided may be main causes of liver failure (4). The golden standard for diagnosis of SALI is the increased total bilirubin (TBIL) or alanine transaminase (ALT) (5). However, neither TBIL nor ALT could early identify the occurrence of liver injury. In addition, Zagory et al. (6) found that the cut-off value of ALT had the possibility of over-predicting low risk liver injury in children with trauma. So, it is necessary to screen clinical or laboratory indexes to develop the sensitive, accurate, and convenient predictors for early identification of SALI.

Platelet (PLT) is involved in sepsis complicated multi-organ dysfunction via regulating inflammation, tissue integrity, and defending against infection (7). Several studies indicated that PLT is a prognostic predictor for sepsis in severe burns (8), and mean platelet volume (MPV) to PLT ratio (MPV/PLT) is a promising predictor of early mortality in severe sepsis (9). In another view, PLT and MPV/PLT are potential surrogate markers predicting liver cirrhosis (10, 11). Moreover, the aspartate aminotransferase (AST) to PLT ratio, named as APRI, was an effective non-invasive assessment of hepatic fibrosis in patients with non-alcoholic fatty liver disease or hepatitis C-related fibrosis (12, 13). Either liver cirrohosis or hepatic fibrosis is closely related to liver dysfunction and early inflammatory stimuli, which share the same characteristic with SALI. We hypothesized that PLT related indexes may provide potential early warning of SALI in children.

In the present study, we conducted a database study based on 4-year medical records of patients with sepsis in a tertiary pediatric hospital. The aim of the study is to evaluate the first serological variables including PLT, MPV/PLT, APRI, etc. as indicators of early warning for the occurrence of SALI in children.

## Materials and Methods

### Patients

We analyzed the sepsis database from the PICU at Shanghai Children's Hospital between July 2014 and June 2018. Patients aged 1 month to <18 years old who were diagnosed with sepsis were screened for inclusion. Sepsis and sepsis-associated organ dysfunction were defined based on the International Pediatric Sepsis consensus conference in 2005 (5). Sepsis-associated neurologic dysfunction was considered when the Glasgow Coma Scale (GCS) score is ≤11. The sepsis-associated acute kidney injury (AKI) was defined based on the Pediatrics RIFLE criteria (2008–2012) as urine output, 0.5 ml/kg/h for >8 h and /or an estimated creatinine clearance (eCCl) decrease of at least 25%; If previous glomerular filtration rate (eGFR) was unavailable, a baseline estimated glomerular filtration rate (eGFR) of 100 ml/min/1.73 m^2^ was assumed (14). SALI was defined by the following conditions: (1) TBIL ≥ 4.0 mg/dL, or (2) ALT > 2 folds upper limit of normal level for age. According to the occurrence of liver injury during hospitalization, patients were divided into the sepsis group and the SALI group. The patients with congenital heart disease, primary liver disease, tumor, heredity metabolic disease, or cardiac arrest were excluded. The patients without complete medical records were excluded. If a patient had multiple admissions to PICU, only the first PICU admission was included. The study was approved by the ethics committee of Children's Hospital affiliated to Shanghai Jiao Tong University (Approval number: 2018R039-F01). Requirement for consent was waived as the data were recorded anonymously in database.

### Observational Variables

Data on all patients admitted to PICU were obtained through a computerized database for all inpatient records at Shanghai Children's Hospital. We collected the clinical parameters including age, sex, co-morbidities, infection sources, and PRISM III score. The laboratory indexes include routine blood test (PLT, MPV, etc.), biochemical indexes for organ functions (direct bilirubin, DBIL; total bilirubin, TBIL; alanine aminotransferase, ALT; AST; γ-glutamyltranspeptidase, γ-GT; lactate dehydrogenase, LDH; blood urea nitrogen, BUN; creatinine, Cr; lactic acid, Lac), coagulation function (prothrombin time, PT; international normalized ratio, INR; fibrinogen, Fib), and infectious indexes (c-reaction protein, CRP; white blood cell, WBC). The outcome variables including length of PICU stay and discharged survival status. The laboratory indexes were collected from the first test within 24 h after PICU admission. If a variable was measured more than once within the first 24 h after PICU admission, the value associated with the severity of illness was selected.

### Statistical Analyses

Data analyses were performed using STATA 15.0 MP (College Station, Texas, USA). Continuous variables were summarized as means ± standard derivations (SD) for normal distribution data and as median (Inter Quartile Range, IQR) for abnormal distribution data. Student *t*-test was used to compare the means of continuous variables with normally distributed data; otherwise, the Mann–Whitney *U*-test was used. The *chi*-square test was used to compare the categorical data. Unadjusted associations between covariates and SALI were estimated by bivariable logistic regression models. Adjusted odd ratios (*ORs*) were estimated by multivariate logistic regression models with inclusion of covariate terms chosen based on the biological plausibility of possible confounding of laboratory indexes and SALI. In order to appreciate the accuracy of independently risk factor as predictor of SALI, a ROC curve was generated. Datasheet for this work was supplied online (Supplementary Table 1). A value of *P* < 0.05 was considered statistically significant. All *P*-values presented are two-tailed.

## Results

**Figure 1:**
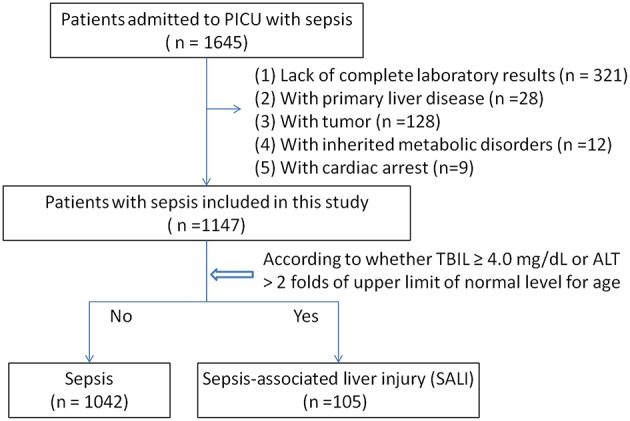
Flowchart of patients included in this study.

Among 1,645 eligible patients, 321 cases were excluded due to lack of complete medical records, and patients with primary liver disease (28 cases), tumor (128 cases), or heredity metabolic disease (12 cases), cardiac arrest (9 cases) were excluded. Leaving 1,147 patients were available for analyses (Figure 1). There were 110 patients with severe sepsis (84 cases in the sepsis group vs. 26 cases in the SALI group) and 151 patients with septic shock (125 cases in the sepsis group vs. 26 cases in the SALI group). The incidence rate of severe sepsis or septic shock was significantly higher in the SALI group compared with the sepsis group (24.76 vs. 8.06 %, *P* < 0.001; 24.76 vs. 12.0 %, *P* < 0.001, respectively). Patient characteristics of the study cohort were stratified according to the occurrence of SALI during hospitalization (Table 1).

**Table 1:** Baseline characteristics of patients with sepsis or sepsis-associated liver injury (SALI).

	Total (*n* = 1,147)	Sepsis (*n* = 1,042)	SALI (*n* = 105)	*P*-value
Age, month	28 (10, 62)	29 (10, 62)	18 (7, 48)	0.038
Male, n (%)	691 (60.24)	629 (60.36)	62 (59.05)	0.793
PRISM III	8 (5, 9)	8 (5, 8)	10 (7, 14)	<0.001
Length of PICU stay, day	10 (6, 17)	10 (6, 17)	13 (8, 22)	0.010
Severe sepsis, n (%)	110 (9.59)	84 (8.06)	26 (24.76)	< 0.001
Septic shock, *n* (%)	151(13.17)	125 (12.0)	26 (24.76)	< 0.001
**Complications**				
Respiratory failure, *n* (%)	321 (27.99)	293 (28.12)	28 (26.67)	0.752
Acute kidney injury, *n* (%)	40 (3.49)	38 (3.65)	2 (1.90)	0.354
Neurologic dysfunction, *n* (%)	47 (4.10)	40 (3.84)	7 (6.67)	0.164
Gastrointestinal, *n* (%)	42 (3.66)	34 (3.24)	8 (7.62)	0.024
**Number of organ dysfunction**, ***n*** **(%)**				
0	764 (66.61)	693 (66.51)	71 (67.62)	
1	324 (28.25)	297 (28.50)	27 (25.71)	0.613
2	51 (4.45)	48 (4.61)	3 (2.86)	0.416
3	8 (0.70)	4 (0.38)	4 (3.81)	0.002
Hospital mortality, *n* (%)	107 (9.33)	82 (7.87)	25 (23.81)	<0.001

## Baseline Characteristics of Patients

In the study cohort, the median (IQR) age of patients at PICU admission was 69 (24, 154) months. Most patients were male (60.24%) in both sepsis and SALI groups. The incidence of SALI was 9.15 % (105/1147). The median (IQR) length of PICU stay was 13 (8, 15) days in patients with SALI and 10 (6, 16) days in patients with sepsis (*P* = 0.010). In the study cohort, the hospital mortality rate of patients with SALI was 23.81 % (25/105), which was significantly higher than that in patients with sepsis (7.87 %, [82/1042]) (*P* < 0.001). The incidence rate of gastrointestinal dysfunction (*P* = 0.024) and more than 3 organs dysfunction (*P* = 0.002) on PICU admission were significantly higher in the SALI group compared with the sepsis group (Table 1). There was no significant different in aspects of age, gender, rate of respiratory failure, AKI, or neurologic dysfunction, rate of <3 organ dysfunction (all *P* > 0.05).

### Multivariate Logistic Regression Analysis for Risk Factors of SALI Occurrence

**Figure 2:**
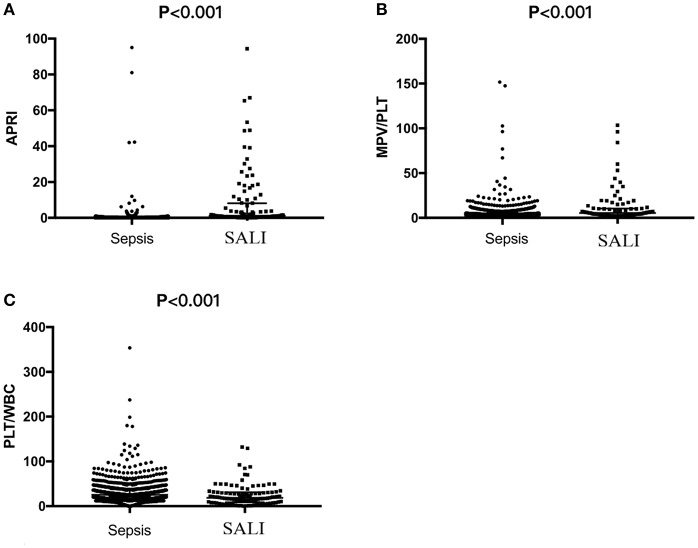
The values of APRI, MPV/PLT or PLT/WBC based on the first serological parameters within 24 h after PICU admission in pediatric patients with sepsis or sepsis-associated liver injury (SALI). **(A)** APRI, **(B)** MPV/PLT, and **(C)** PLT/WBC.

Laboratory indexes of the study cohort were stratified according to whether the patients were complicated by SALI during hospitalization (Table 2). Besides of the classic of indexes for liver injury including DBIL, TBIL, ALT, AST, γ-GT and LDH, the indicators for coagulation including PT and INR were significantly increased (all *P* < 0.05), but the levels of Fib were significantly decreased in SALI group compared with sepsis group (*P* < 0.001) (Table 2). In addition, the levels of BUN and lactate (Lac) were significantly higher in the SALI group compared with the sepsis group (*P* = 0.017, *P* = 0.007, respectively). The value of PLT-related combined indexes including PLT and MPV were significantly different between SALI and sepsis groups (183 [65, 312] vs. 274 [192, 370], *P* < 0.001; 11.1 [10.1, 11.8] vs. 10.2 [9.5, 110], *P* < 0.001, respectively) (Table 2). In addition, the values for APRI, MPV/PLT, and PLT/WBC was 1.20 [0.46, 8.21] vs. 0.13 [0.08, 0.22], *P* < 0.001; 0.054 [0.033, 0.106] vs. 0.038 [0.027, 0.057], *P* < 0.001; 18.95 [9.33, 31.12] vs. 25.49 [16.41, 37.42], *P* < 0.001, respectively) (Figure 2). The values of APRI was positively correlated with the standard diagnostic indexes of ALT and TBIL in our study cohort (*r* = 0.477, *P* < 0.001; *r* = 0.269, *P* < 0.001, respectively). However, there was no correlation either MPV/PLT or PLT/WBC with ALT or TBIL (all *P* > 0.05).

**Table 2:** The laboratory indexes of patients with sepsis or sepsis-associated liver injury (SALI).

	Total (*n* = 1,147)	Sepsis (*n* = 1,042)	SALI (*n* = 105)	*P*-value	Unadjusted *OR* (95% *CI*)
**Metabolic indexes**					
DBIL (μmol/L)	2.14 (1.38, 3.64)	2.10 (1.36, 3.43)	4.09 (1.82, 27.68)	<0.001	1.092 (1.060–1.126)
TBIL (μmol/L)	7.70 (4.97, 11.58)	7.44 (4.86, 11.17)	10.41 (6.28, 35.57)	<0.001	1.061 (1.044–1.079)
**Indicators for liver function**					
ALT (U/L)	21 (14, 33)	20 (13, 29)	151 (101, 347)	<0.001	1.111 (1.086–1.135)
AST (U/L)	36 (27, 54)	35 (27, 47)	200 (104, 472)	<0.001	1.028 (1.023–1.033)
γ-GT (U/L)	14 (11, 25)	14 (10, 21)	65 (28, 161)	<0.001	1.018 (1.014–1.022)
LDH (U/L)	507 (335, 813)	481 (325, 781)	884 (466, 1623)	<0.001	1.001 (1.001–1.002)
**Indicators for coagulation function**					
PT (s)	12.7 (11.8, 13.9)	12.7 (11.7, 13.7)	13.3 (11.9, 15.8)	0.002	1.090 (1.041–1.141)
INR	1.12 (1.04, 1.22)	1.12 (1.04, 1.21)	1.17 (1.05, 1.38)	0.002	2.742 (1.646–4.566)
Fib (g/L)	3.10 (2.18, 4.24)	3.20 (2.27, 4.25)	2.31 (1.46, 3.41)	<0.001	0.656 (0.548–0.785)
**Platelet**					
PLT (× 10^9^/L)	266 (179, 367)	274 (192, 370)	183 (65, 312)	< 0.001	0.995 (0.993–0.997)
MPV	10.3 (9.6, 11.1)	10.2 (9.5, 11.0)	11.1 (10.1, 11.8)	< 0.001	1.588 (1.335–1.888)
**Indicators for kidney function**					
BUN (mmol/L)	3.3 (2.4, 4.4)	3.3 (2.4, 4.3)	3.8 (2.4, 6.3)	0.017	1.046 (1.009–1.085)
Cr (μmol/L)	26 (21, 34)	26 (21, 33)	27 (20, 37)	0.472	0.999 (0.998–1.002)
**Infection indicator**					
WBC (× 10^9^/L)	10.25 (6.49, 16.03)	10.26 (6.58, 16.13)	9.09 (5.21, 15.34)	0.100	0.976 (0.947–1.006)
CRP (mg/L)	48 (17, 99)	49 (18, 100)	42 (13, 88)	0.079	0.996 (0.992–1.001)
**Indicator for hemodynamics**					
Lac (mmol/L)	1.7 (1.1, 2.6)	1.6 (1.1, 2.5)	1.9 (1.2, 3.2)	0.007	1.200 (1.106–1.303)

Multivariate logistic regression analysis indicated that the independent variables related to SALI occurrence included APRI (*OR*: 1.065, 95% *CI*: 1.031–1.101), γ-GT (*OR*: 1.015, 95% *CI*: 1.011–1.020), and LDH (*OR*: 1.001, 95% *CI*: 1.001–1.001) when adjusted by PT, INR, Fib, Lac, CRP, MPV. However, MPV/PLT and PLT/WBC were not independent factors of SALI occurrence (Table 3). The *OR* of SALI occurrence in the APRI ≥ 0.340 group was 16.571-fold that of those with APRI < 0.340 as referent adjusted by age, gender, PT, INR, Fib, LDH, γ-GT, Lac, CRP, MPV (Table 3).

**Table 3:** Multivariate logistic regression analysis of risk factors for sepsis-associated liver injury (SALI).

Variables	Unadjusted *OR* (95% *CI*)	*P*-value	Adjusted *OR* (95% *CI)*	*P*-value
**Model 1**				
APRI	1.116 (1.073–1.160)	<0.001	1.065 (1.031–1.101)	0.010
APRI ≥ 0.340	22.990 (13.212–40.001)	<0.001	16.571 (7.279–37.726)	<0.001
APRI < 0.340	1.000	Referent	1.000	Referent
γ-GT	1.018 (1.014–1.022)	<0.001	1.015 (1.011–1.020)	<0.001
LDH	1.001 (1.001–1.002)	<0.001	1.001 (1.001–1.001)	<0.001
**Model 2**				
MPV/PLT	1.333 (1.009–1.762)	<0.001	–	–
**Model 3**				
PLT/WBC	0.986 (0.974–0.998)	0.001	–	–

### ROC Analysis for Variables as Biomarkers for Early Warning of SALI Occurrence

**Figure 3:**
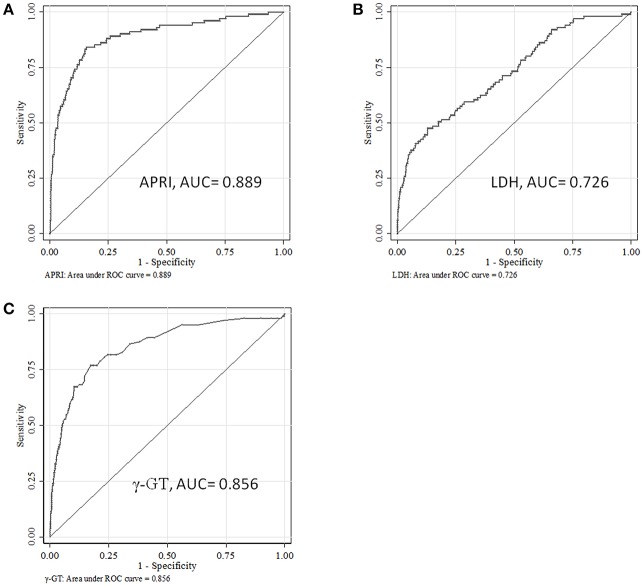
The ROC curves for APRI, LDH or γ-GT to predict the occurrence of sepsis-associated liver injury (SALI). **(A)** APRI, **(B)** LDH, and **(C)** γ-GT.

The area under the ROC curve (AUC) of APRI, γ-GT and LDH for the prediction model for SALI occurrence was 0.889 (95 % *CI*: 0.851–0.927), 0.856 (95 % *CI*: 0.814–0.897), and 0.726 (95 % *CI*: 0.671–0.780), respectively (Figure 3). The cutoff point of ARPI was 0.340 with a clinical sensitivity 84.6 % and a specificity of 84.3 % for prediction of SALI. In addition, the sensitivity of γ-GT at 27 U/L or LDH at 966 U/L for predicting SALI was 76.9 and 47.5%, and the specificity of γ-GT at 27 U/L or LDH at 966 U/L for prediction of SALI was 82.6 and 87.1%, respectively (Table 4). The AUC of APRI was superior to that of LDH (*P* < 0.001), and it was not inferior to that of γ-GT (*P* = 0.272) (Table 4).

**Table 4:** ROC analysis about laboratory indexes for predicting the occurrence of sepsis-associated liver injury (SALI).

Variables	AUC (95% CI)	Cutoff	Sensitivity (%)	Specificity (%)	*P*-value
APRI	0.889 (0.851–0.927)	0.340	84.6	84.3	
LDH	0.726 (0.671–0.780)	966	47.5	87.1	<0.001
γ-GT	0.856 (0.814–0.897)	27	76.9	82.6	0.272

## Discussion

Early recognition of SALI is essential for timely management for patients with sepsis to prevent the progress to MODS, which is still challenge in PICU. In this study, we analyzed the database regarding clinical and laboratory indexes based on 4-years medical records of pediatric patients with sepsis. To our knowledge, this is the first report that APRI is an early predictor of warning for SALI occurrence.

The incidence of pediatric SALI was estimated range to be from 1.3 to 46.6 % depending on the restrictiveness of the definition (17), probably due to lack of diagnostic tools, notably those that can detect the early liver insult (4). In our present study, the incidence of SALI in pediatric patients was 9.15 % (105/1,147), and hospital mortality of pediatric patients with SALI was 23.81 % (25/105). The higher hospital mortality in the SALI group suggested that SALI is correlated with poor prognosis of sepsis, which was consistent with previous study (18). Several reports indicated that the incidence of SALI in adult was 34.7% (156/449) (16), and the mortality of adult patients with sepsis complicated by serum TBIL >2.0 mg/dL was 42% (19). According to our present results, the incidence and mortality of SALI in pediatric patients was relatively lower than that of adult patients. Considering that age affects the assessment of liver injury (20), it is essential to investigate specific predictors for early identification of liver insult in pediatric patients with sepsis, instead of using the experience of adult's clinical study for reference.

Though biomarkers for early diagnosis of liver injury were reported based on animal and clinical study, like glutamate dehydrogenase (GLDH), total cytokeratin 18 (K18), caspase cleaved K18, etc. (21), it is still crucial for the clinicians to make decision based on the results of routine clinical and laboratory test. PLT is involved in hepatic response to early sepsis and plays a key role in hepatocyte injury via interacting with neutrophils, Kuffer cells, and endothelial cell (22). Platelet aggregation is retained in the liver sinusoids causing liver dysfunction (23). Platelet consumption results in lower level of PLT in circulation. Moreover, PLT and MPV/PLT were proved to be promising predictors of early mortality in sepsis (8, 9). In another aspect, AST is an enzyme, primarily existing in hepatocytes. Intracellular AST is released into the systemic circulation when hepatocytes are inevitably injured. Currently, AST is a widely accepted clinical marker for liver injury. Serum AST levels can be influenced by myocardial damage and renal injury (15). Patients with cardiac injury were excluded in this study. The levels of AST were no significant difference between the sepsis group and the sepsis-associated AKI group, which suggested that the levels of AST could not be affected by the complication of AKI (data not shown). In the present study, the combined index of APRI has the best effect on early warning for SALI, which was superior to LDH and not inferior to γ-GT. APRI had powerful value for predicting the occurrence of SALI with a specificity of 84.3% and a sensitivity of 84.6 %. Liver injury occurs at early phase of severe sepsis, which is characterized by cholestasis, steatosis, hepatocellular injury, impaired regeneration, a decreased response to the cytokine interleukin-6, and high mortality (24). Recently, Vongbhavit et al proved that APRI can effectively predict the occurrence of parenteral nutrition-associated cholestasis in premature infants with intestinal perforation (25). Whether APRI as an early biomarker for warning of SALI is related to sepsis-induced cholestasis or steatosis needs to investigate in the future. In addition, Diaz et al proved that APRI was positively correlated with TBIL, ALT and INR, and negatively correlated with ALB (26). Consistently, the values of APRI were positively correlated with the values of TBIL and ALT in our present study. We speculated that APRI might be used to evaluate the status of hepatic metabolism and coagulation function. The mechanisms underlying APRI involved in SALI need further investigation.

There were several limitations in our present study. It was a database study. The retrospective study design might contribute the lower incidence of AKI and brain dysfunction due to recording bias. Otherwise, the data were analyzed based on the medical records from single PICU, though the sample size was big with a long study period. Though we collected the parameters within 24 h after PICU admission as potential early predictors, the changes of interested variables were lack in the present study, for example PRISM III score, lactate, *etc*. If available, we would enable a well-designed, prospective study to assess the role of APRI in SALI in the future.

## Conclusions

To our knowledge, this is the first report that APRI is an early predictor for warning of SALI in pediatric patients with sepsis. The elevated APRI (≥ 0.340) within 24 h after PICU admission is a valuable predictor for higher risk for the occurrence of SALI.

## Lay Summary

Early warning and appropriate treatment of liver injury is critical for recovery in patients with sepsis. AST-to-platelet ratio index (APRI) is an independent risk factor of sepsis-associated liver injury (SALI) occurrence, and elevated APRI within 24 h after admission (>0.340) is a potential predictor for SALI in children.

## Author Contributions

CW and YZha conceived and designed the study. JD, YZho, and MC collected and analyzed data. JD, CW, YC, and YZha contributed analysis tools and discussion. JD, CW, and YZha wrote the paper.

### Conflict of Interest Statement

The authors declare that the research was conducted in the absence of any commercial or financial relationships that could be construed as a potential conflict of interest.

## Abbreviations

ALT: Alanine aminotransferase
AST: Aspartate aminotransferase
BUN: Blood urea nitrogen
Cr: Creatinine
CRP: C-reaction protein
DBIL: Direct bilirubin
Fib: Fibrinogen
γ-GT: γ-glutamyl transpeptidase
INR: International normalized ratio
Lac: Lactate
LDH: Lactate dehydrogenase
MPV: Mean platelet volume
PICU: Pediatric intensive care unit
PLT: Platelet
PRISM III: Pediatric Risk of Mortality III
PT: Prothrombin time
TBIL: Total bilirubin
WBC: White blood cell.
